# Supplementary figures and images for: Korean Red Ginseng Improves Astrocytic Mitochondrial Function by Upregulating HO-1-Mediated AMPKα–PGC-1α–ERRα Circuit after Traumatic Brain Injury
Source: Int J Mol Sci. 2021 Dec 3;22(23):13081. doi: 10.3390/ijms222313081 (PMC8657744; doi:10.3390/ijms222313081)

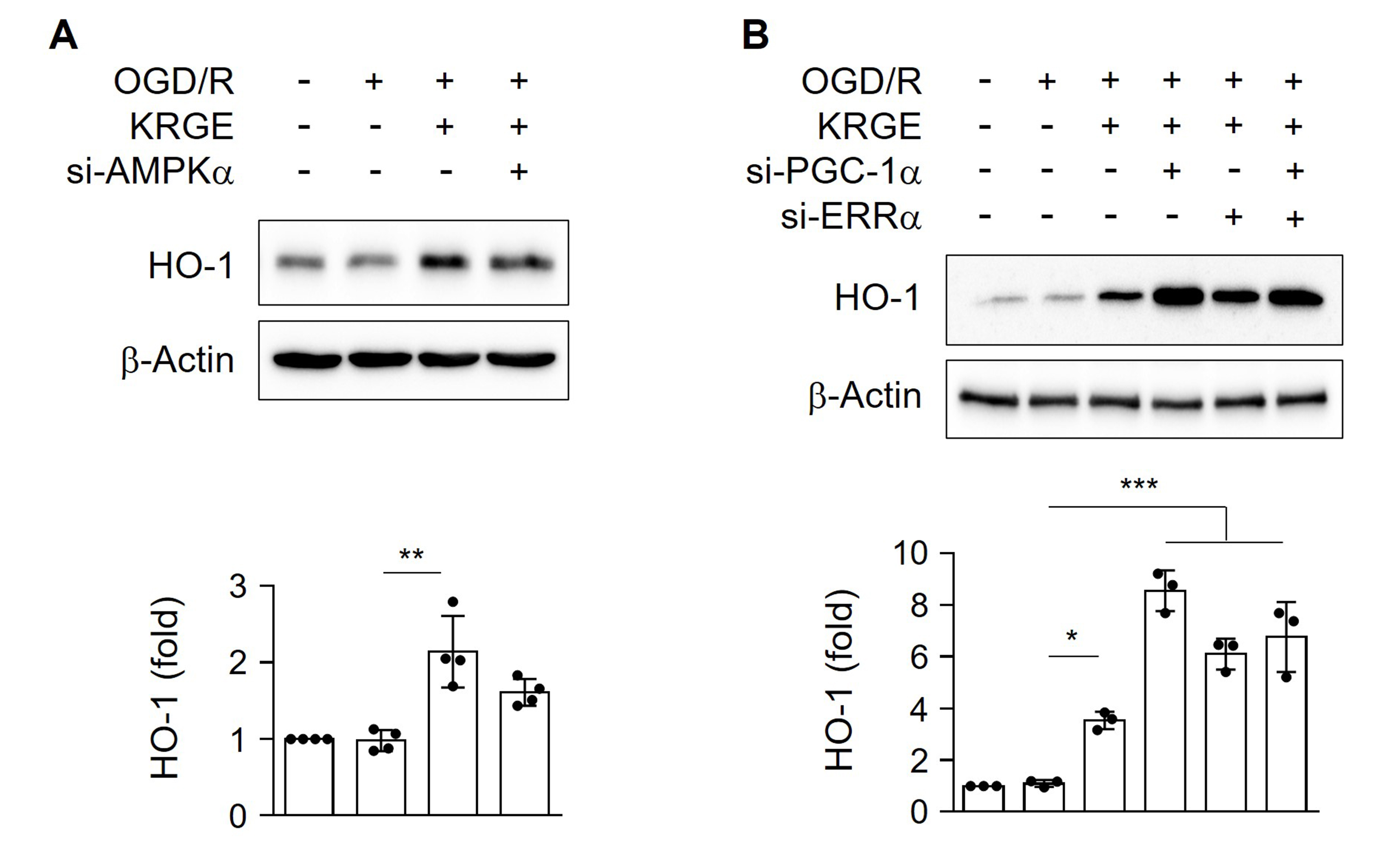

Supplement: Supplementary file 1 [file ijms-22-13081-s001.zip › ijms-1457294-supplementary.jpg]
